# Spiked Helmet Sign Associated With Acute Gastric Distension and Hemodynamic Instability: A Case Report

**DOI:** 10.7759/cureus.115918

**Published:** 2026-09-07

**Authors:** Ricardo Gonzalez

**Affiliations:** 1 Faculty of Medicine, Universidad del Desarrollo, Santiago, CHL

**Keywords:** critical care, critical illness, electrocardiography, emergency medicine imaging, septic shock, spiked helmet sign

## Abstract

The spiked helmet sign is an uncommon pseudo-ST-segment elevation pattern reported in critical illness and recognized by an upward baseline shift beginning before the QRS complex and a dome-and-spike contour. Its distinction from acute coronary occlusion is clinically important but can be difficult when myocardial injury, left ventricular hypertrophy, electrolyte abnormalities, or post-resuscitation changes coexist. We report a 34-year-old male with end-stage kidney disease on hemodialysis, hypertension, hypertrophic cardiomyopathy, and thrombotic microangiopathy who developed aspiration-related respiratory failure, peri-intubation asystolic cardiac arrest, septic shock, and marked gastric distension. During peak instability, an electrocardiogram showed sinus tachycardia at 135 beats/min, left ventricular hypertrophy with secondary repolarization abnormalities, and a spike-helmet morphology that was most prominent in lead II and less distinct in V3. Urgent nasogastric decompression evacuated gas and gastric contents. A repeat tracing obtained 20 minutes later showed a lower heart rate and partial attenuation of the dynamic morphology. A late tracing, obtained after resolution of gastric distension and hemodynamic stabilization, showed sinus rhythm at 83 beats/min and disappearance of the pre-QRS dome-and-spike configuration in lead II and V3, while baseline hypertrophy-related repolarization abnormalities persisted. The serial relationship supports a reversible extracardiac contribution, but incomplete serial biomarkers, echocardiographic assessment, intra-abdominal pressure measurements, and electrode-position documentation prevent definitive exclusion of concurrent myocardial injury or artifact. This case emphasizes integrating electrocardiographic morphology with bedside physiology, abdominal examination, imaging, and response to reversible interventions while continuing to evaluate for true myocardial ischemia.

## Introduction

The spiked helmet sign is a rare electrocardiographic pattern first named in 2011 after its resemblance to the Pickelhaube, the historical Prussian spiked helmet. The classic description is an apparent ST-segment elevation with an upward baseline shift that begins before the QRS complex, producing a dome-and-spike contour rather than conventional ST elevation measured from a stable isoelectric baseline [1,2].

Recognition matters because the pattern can mimic acute coronary occlusion while occurring during severe noncardiac illness. Reported associations include sepsis, respiratory failure, intracranial hemorrhage, pneumothorax, acute abdominal disease, and other states of major physiologic stress. Proposed mechanisms include changes in intrathoracic or intra-abdominal pressure, diaphragmatic or body-wall motion, electromechanical artifact, sympathetic activation, prolonged repolarization, and macroscopic T-wave alternans [2-5].

Published evidence linking the spiked helmet sign to abdominal pathology remains limited to case reports and small case series, including reports involving bowel obstruction, ileus, and gastric dilatation. Therefore, the present case should be interpreted as an additional temporal observation rather than proof of a mechanism. We report a critically ill patient with marked gastric distension and spiked helmet morphology most prominently expressed in lead II, with lesser involvement of V3. The case adds a short-interval post-decompression electrocardiogram that showed persistence with partial attenuation, followed by a later tracing after clinical stabilization that separated the dynamic pre-QRS component from fixed hypertrophy-related ST-T abnormalities.

## Case presentation

A 34-year-old male with end-stage kidney disease requiring chronic hemodialysis, hypertension, hypertrophic cardiomyopathy, and thrombotic microangiopathy presented with acute oppressive abdominal pain, nausea, and vomiting. Reported systolic blood pressure reached 260 mmHg, and the abdominal examination suggested peritoneal irritation.

During hospitalization, he developed abrupt neurologic deterioration to a Glasgow Coma Scale score of 6 and bronchoaspiration. Endotracheal intubation was followed by an asystolic cardiac arrest attributed clinically to hypoxia. Return of spontaneous circulation was achieved after the second cycle of advanced resuscitation.

On transfer to the intensive care unit, shock persisted despite norepinephrine at 0.4 mcg/kg/min and epinephrine at 0.2 mcg/kg/min. Available studies documented multiorgan dysfunction, severe metabolic acidosis, elevated inflammatory biomarkers, and a high-sensitivity troponin T concentration of approximately 200 ng/L. This troponin result was available as a single value in the source record reviewed for manuscript preparation; the assay reference range and serial trend were not available. In the setting of end-stage kidney disease, vomiting, severe acidosis, shock, and recent asystolic arrest, this isolated elevation was not specific for acute coronary occlusion. Potassium, calcium, magnesium, and bicarbonate values aligned to each of the three electrocardiograms were not available in the reviewed source record; therefore, an electrolyte-driven contribution to the repolarization abnormalities could not be excluded. Computed tomography angiography of the chest, abdomen, and pelvis showed cardiomegaly and segmental inflammatory thickening of the small bowel wall. Marked gastric distension was evident clinically and on the computed tomography angiography scout image (Figure 1).

**Figure 1:**
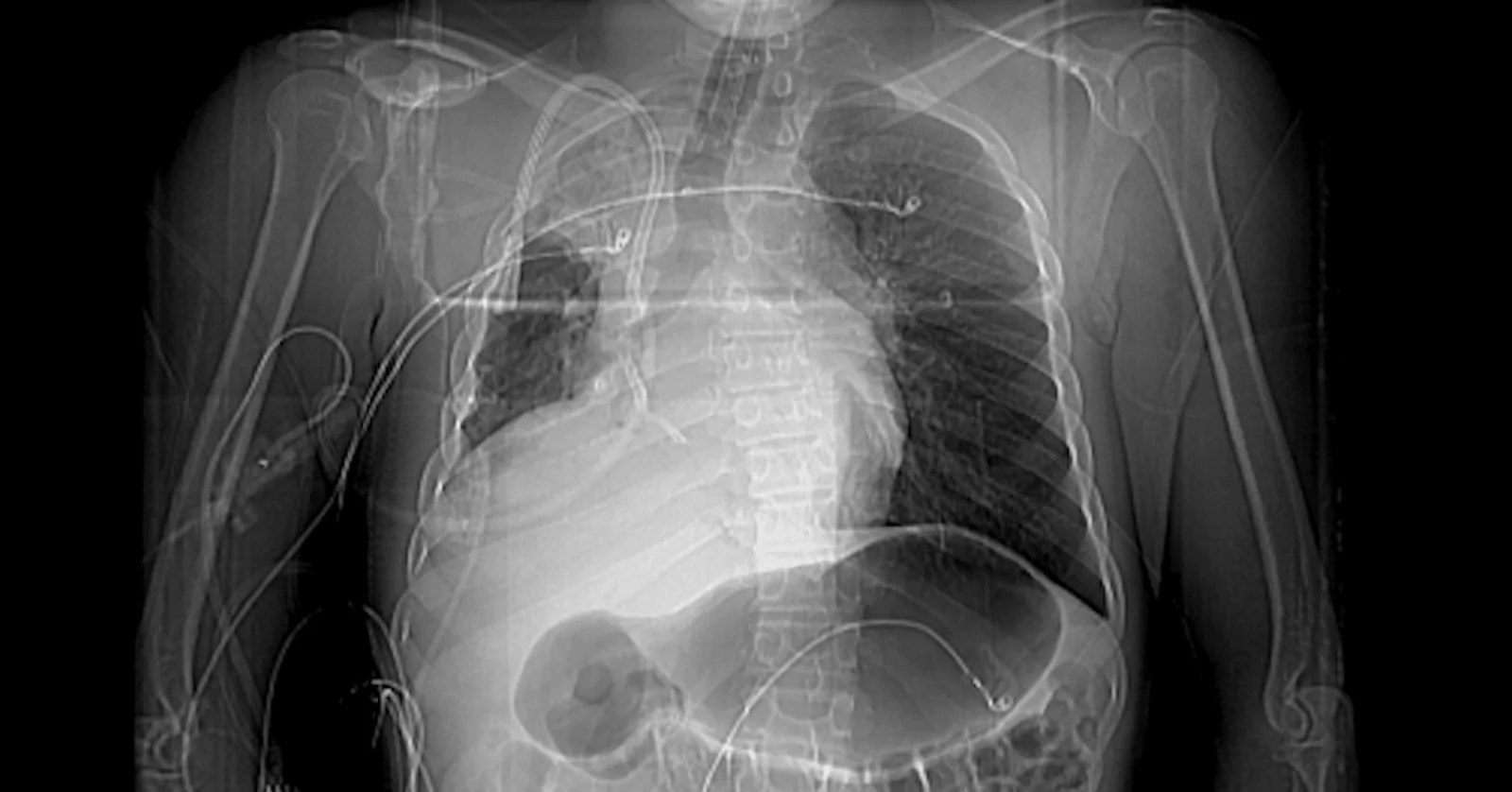
Anteroposterior scout image. Anteroposterior scout image acquired during computed tomography angiography of the chest, abdomen, and pelvis, showing marked air-filled gastric distension beneath the left hemidiaphragm. The finding was concordant with the abdominal examination and supported urgent gastric decompression.

The electrocardiographic sequence was revised according to the corrected clinical chronology and the heart rates printed on the tracings. Original ECG-machine timestamps and complete image metadata sufficient for exact timestamp reconstruction were not available in the source record reviewed; therefore, the interval between the initial and second tracing is reported as approximate. Heart rate is reported as a feature of each tracing and was not used alone as proof of physiologic improvement. The first tracing was recorded during severe shock and maximal gastric distension (Figure 2). It showed sinus tachycardia at 135 beats/min, narrow QRS complexes, and left ventricular hypertrophy with secondary repolarization abnormalities. The most prominent spiked helmet morphology was present in lead II, where a progressive upward baseline shift began before the QRS complex, formed a dome-like contour, and was interrupted by a tall QRS complex protruding as the spike. A similar, although less distinct, morphology was observed in V3. The coexistence of myocardial injury, hypertrophy, unavailable aligned electrolyte values, and recent cardiac arrest prevented ischemia from being excluded by morphology alone.

**Figure 2:**
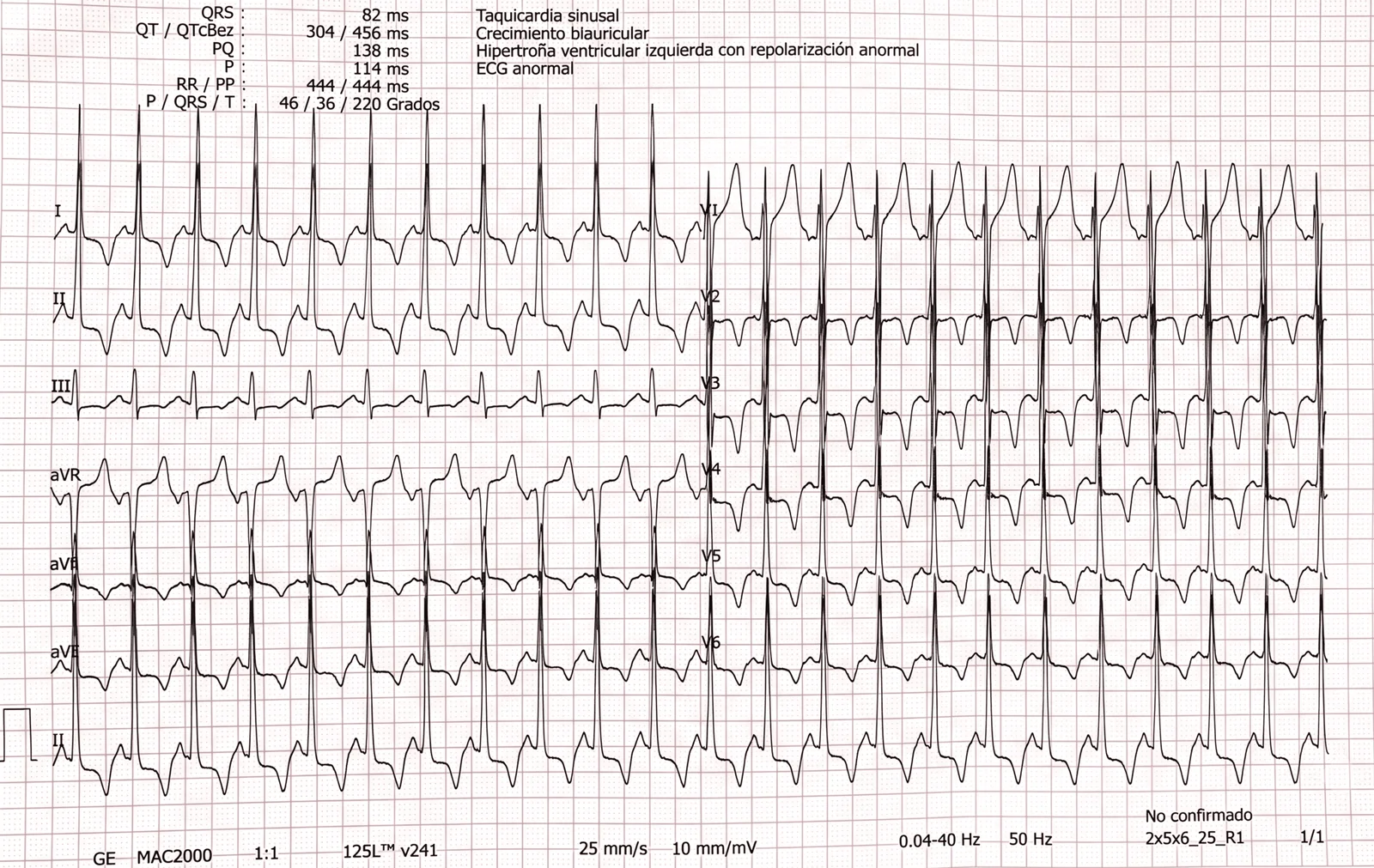
Initial electrocardiogram. Initial electrocardiogram during marked gastric distension and severe hemodynamic compromise. The de-identified tracing shows sinus tachycardia at 135 beats/min, left ventricular hypertrophy with secondary repolarization abnormalities, and spiked helmet morphology most prominent in lead II and less distinct in V3. Automated measurements printed on the tracing include a QRS of 82 ms and QT/QTc of 304/456 ms. These automated QT/QTc values are reported as displayed by the ECG machine and are not used for mechanistic interpretation because the abnormal baseline morphology limits automated interval measurement.

Urgent nasogastric decompression evacuated gas and gastric contents and reduced the abdominal distension. The exact output volume and character were not available in the reviewed source record. Bladder-pressure measurement or another quantified intra-abdominal pressure measurement was not available. A second electrocardiogram obtained approximately 20 minutes after the initial tracing and shortly after decompression showed sinus tachycardia at 119 beats/min and persistence with partial attenuation, rather than complete disappearance, of the pre-QRS dome-and-spike morphology in leads II and V3 (Figure 3). Immediate paired hemodynamic measurements, ventilatory mechanics, and abdominal-pressure data were not available.

**Figure 3:**
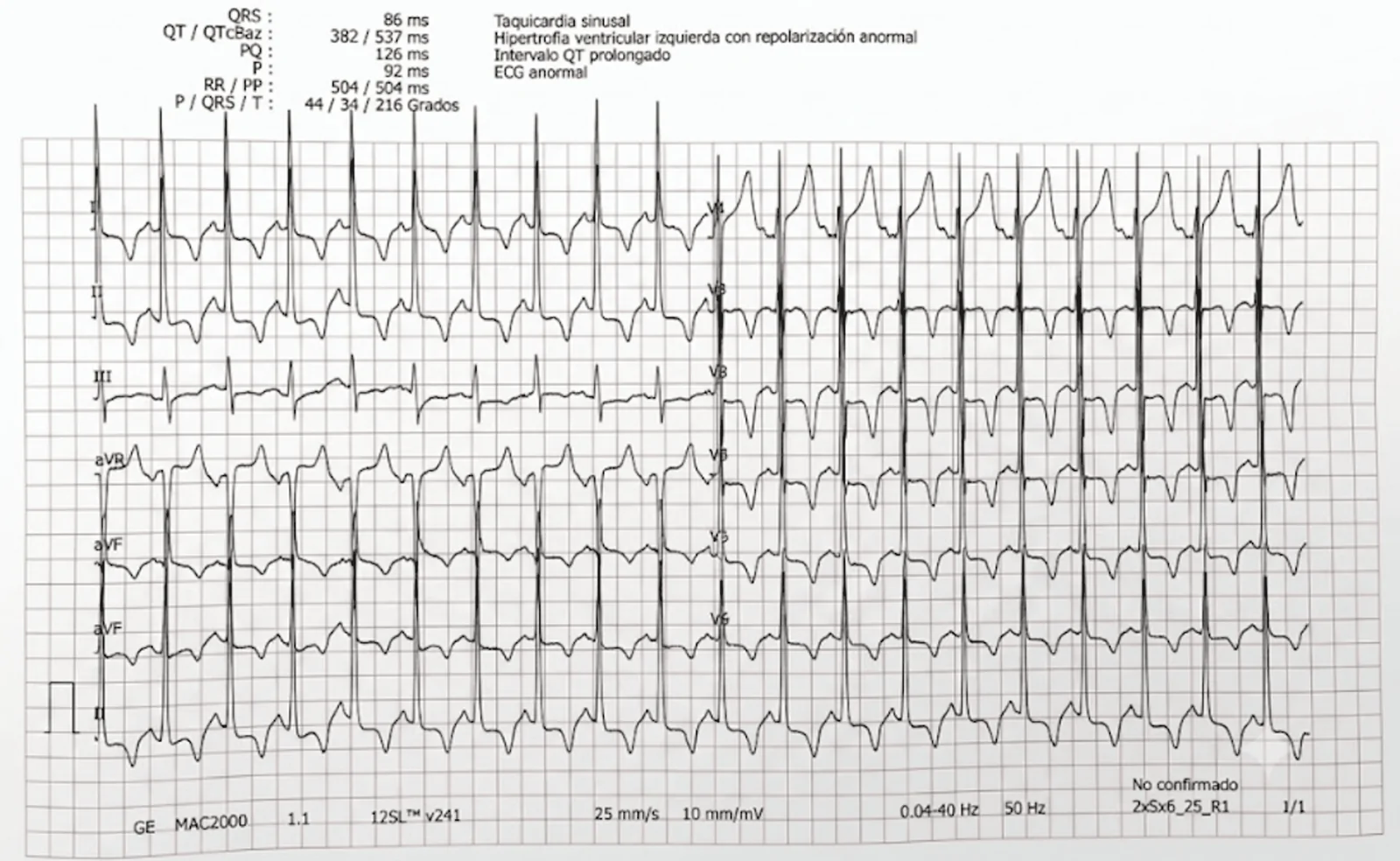
Early post-decompression electrocardiogram. Early post-decompression electrocardiogram obtained approximately 20 minutes after the initial tracing and shortly after gastric decompression. The exact interval from completion of decompression to this tracing was not documented. The de-identified tracing shows sinus tachycardia at 119 beats/min, with persistent but partially attenuated pre-QRS dome-and-spike morphology in leads II and V3. Automated measurements printed on the tracing include a QRS of 86 ms and QT/QTc of 382/537 ms; these are reported only as machine-printed values and are not interpreted because baseline distortion limits automated QT/QTc assessment.

Over the following days, the aspiration-related respiratory infection, septic shock, and abdominal distension improved, and vasopressor support was progressively reduced. Other co-occurring critical-care interventions also occurred during this multiday interval. At the latest documented intensive care follow-up, an electrocardiogram showed sinus rhythm at 83 beats/min. The pre-QRS dome-and-spike configuration was no longer evident in leads II or V3, while the pre-existing left ventricular hypertrophy-related repolarization pattern remained (Figure 4). As the late tracing occurred after multiday stabilization rather than immediately after decompression, the disappearance of the morphology cannot be attributed to gastric decompression alone.

**Figure 4:**
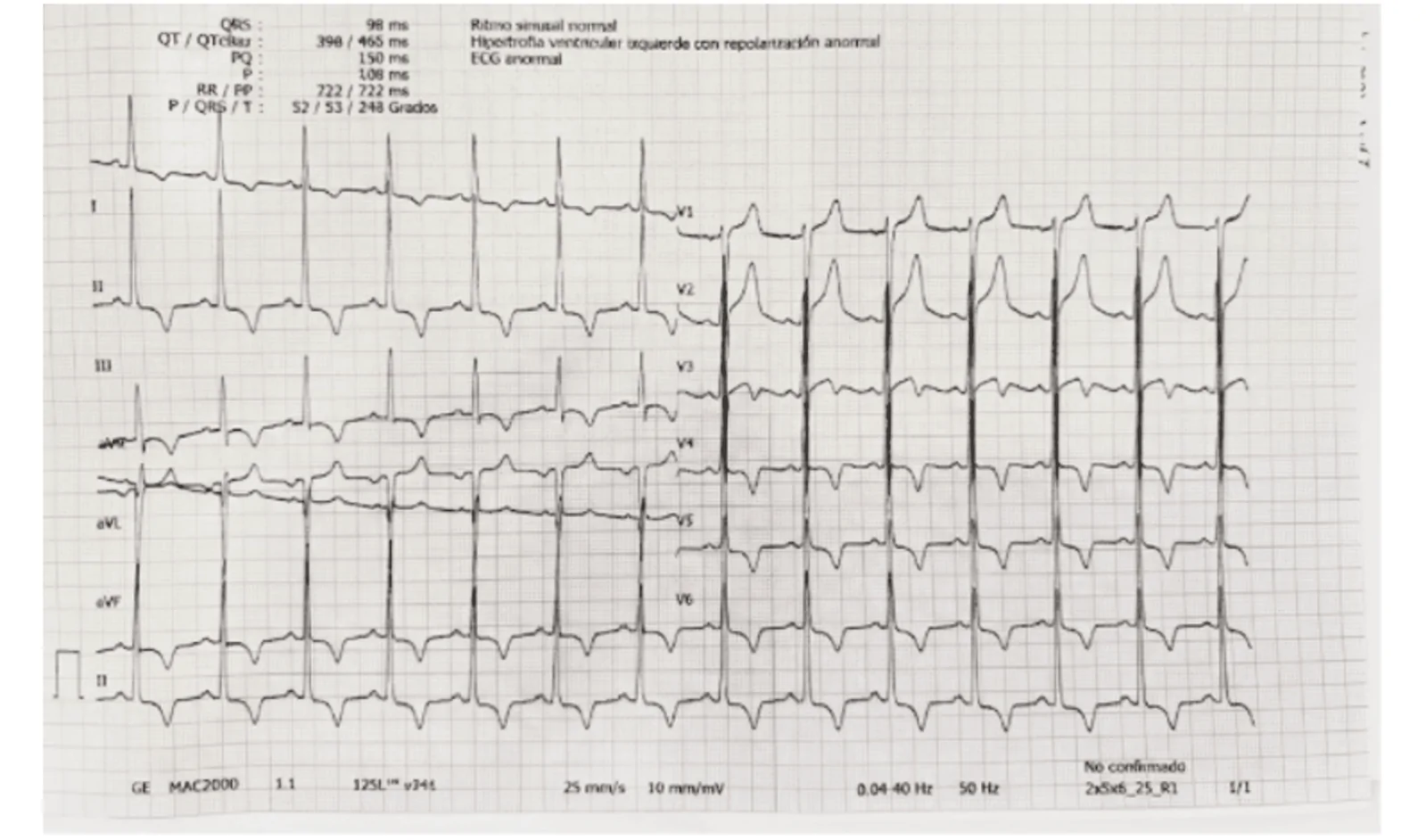
Late follow-up electrocardiogram. Late follow-up electrocardiogram after multiday improvement in gastric distension and hemodynamic instability. Sinus rhythm is present at 83 beats/min. The pre-QRS dome-and-spike configuration is no longer evident in leads II or V3, while left ventricular hypertrophy-related repolarization abnormalities persist. Automated measurements printed on the tracing include a QRS of 98 ms and QT/QTc of 398/465 ms.

The clinical and electrocardiographic timeline of the patient is presented in Table 1.

**Table 1:** Clinical and electrocardiographic timeline.

Clinical time point	Key clinical event	Electrocardiographic or therapeutic interpretation
Presentation	Acute abdominal pain, nausea, vomiting, severe hypertension, and peritoneal irritation.	No spiked helmet interpretation assigned at this stage.
Clinical deterioration	Glasgow Coma Scale score of 6, bronchoaspiration, intubation, asystolic arrest, and return of spontaneous circulation after two cycles.	The post-resuscitation setting created multiple potential causes of dynamic ST-T abnormalities.
T0: peak ICU instability/before gastric decompression	Shock requiring norepinephrine 0.4 mcg/kg/min and epinephrine 0.2 mcg/kg/min, with marked gastric distension.	Initial ECG: sinus tachycardia at 135 beats/min, left ventricular hypertrophy, and spiked helmet morphology most prominent in lead II and less distinct in V3.
Urgent intervention	Nasogastric decompression with evacuation of gas and gastric contents; exact output volume was not available in the reviewed source record.	Intervention directed at a potentially reversible mechanical stressor; bladder pressure or intra-abdominal pressure measurement was not available.
T0 + approximately 20 minutes from the initial ECG; shortly after decompression	Reduced abdominal distension was described clinically. The exact interval from completion of decompression to the second ECG was not documented.	Second ECG: sinus tachycardia at 119 beats/min with persistent but partially attenuated pre-QRS dome-and-spike morphology.
Late intensive care follow-up	Improvement in septic shock and gastric distension with progressive vasopressor reduction.	Third tracing: sinus rhythm at 83 beats/min, persistent hypertrophy-related repolarization changes, and absence of the dynamic pre-QRS dome-and-spike component. Attribution to decompression alone is not possible.

## Discussion

In an unstable patient with pseudo-ST-segment elevation, the differential diagnosis is broad and usually weighted toward cardiac causes, whereas an abdominal contributor may be overlooked early. This case is reported because marked gastric distension was evident at the bedside and treated urgently, creating an opportunity to compare electrocardiographic morphology before decompression, shortly afterward, and after multiday stabilization.

The key observation is persistence rather than immediate normalization. The initial tracing, obtained during peak instability, showed sinus tachycardia at 135 beats/min, left ventricular hypertrophy with secondary repolarization abnormalities, and spiked helmet morphology most prominent in lead II and less distinct in V3. The early post-decompression tracing, obtained approximately 20 minutes after the initial ECG and shortly after gastric decompression, still showed the pre-QRS dome-and-spike morphology, although partially attenuated. Complete disappearance was documented only on late ICU follow-up after a multiday interval that included improvement in septic shock and gastric distension, vasopressor weaning, and other co-occurring critical-care interventions. Therefore, attribution of late ECG normalization to gastric decompression alone is not possible.

Diagnostic restraint is necessary. Marked left ventricular hypertrophy, recent asystolic arrest, severe metabolic stress, and unavailable aligned electrolyte values complicated separation of the transient morphology from the baseline electrocardiographic pattern. With a single elevated high-sensitivity troponin T value after cardiac arrest, no serial troponin kinetics, no available echocardiographic wall-motion assessment, and no available coronary anatomy in the reviewed source record, acute coronary syndrome or acute coronary occlusion was not definitively excluded. Arterial pulsation, electrode placement, and other extracardiac artifacts can also reproduce a spiked helmet morphology [6]. The serial changes support a dynamic component, but the sign should not be treated as pathognomonic or as a substitute for evaluating myocardial ischemia.

Several mechanisms could have acted simultaneously, but the available patient-level data do not allow one mechanism to be confirmed. Gastric distension may elevate the diaphragm, increase intra-abdominal pressure, and alter venous return, particularly during positive-pressure ventilation and shock. Mechanical coupling among cardiac motion, the diaphragm, the abdominal wall, and surface electrodes may generate apparent baseline and ST-segment displacement. At the same time, hypoxia, acidosis, sepsis, and exogenous catecholamines may increase sympathetic tone and ventricular repolarization heterogeneity [2,4,5]. However, nasogastric output volume, intra-abdominal pressure, bladder pressure, ventilator mechanics, plateau pressure, venous return surrogates, and immediate paired hemodynamic responses after decompression were not available in the reviewed source record. These missing measurements preclude causal attribution.

The automated QT/QTc values printed on the tracings are reported only for transparency and should not be interpreted as manually confirmed repolarization measurements. Automated QT measurement assumes a definable isoelectric baseline, which is precisely distorted by the pre-QRS dome-and-spike morphology. Baseline distortion, rather than tachycardia alone, is therefore the main limitation of the machine-reported QT/QTc values. Similarly, the QRS durations printed on the three tracings varied from 82 to 86 to 98 ms. In a dialysis-dependent patient, this reinforces the need for aligned electrolyte values, but those values were not available in the source record reviewed for this revision.

The abdominal association is supported by previous reports, although the literature remains limited to case reports and small case series. Spiked helmet morphology has been used as a clue to intra-abdominal pathology in the emergency setting, and gastric or intestinal distension has produced dynamic pseudo-ischemic tracings that improved after decompression or resolution of obstruction [7,8]. A 2025 report specifically described spiked helmet morphology associated with gastric dilatation [9]. The present case does not establish novelty through the association alone. Its contribution is the bedside serial method: comparing tracings during peak instability, shortly after a reversible intervention, and after late stabilization helped separate a dynamic pre-QRS dome-and-spike component from persistent hypertrophy-related ST-T abnormalities.

The practical differential diagnosis remained broad: acute coronary occlusion, post-resuscitation myocardial injury, hypertrophic cardiomyopathy with secondary repolarization changes, electrolyte disturbance associated with kidney failure, stress cardiomyopathy, pericarditis, and artifact. A spiked helmet tracing should therefore prompt two parallel actions. Clinicians should identify and immediately treat reversible extracardiac stressors, including gastric distension, while continuing an appropriate assessment for myocardial ischemia and other dangerous mimics. Pattern recognition must not be used as a reason to withhold indicated coronary evaluation [10].

This report has important limitations. Intra-abdominal pressure, bladder pressure, nasogastric output volume and character, manual QT correction, precise electrode positions, confirmation of unchanged lead placement across tracings, complete serial electrolyte measurements aligned to the three ECGs, arterial blood gas trends, serial troponin kinetics, echocardiographic wall-motion findings, and coronary anatomy were unavailable in the source record reviewed for manuscript preparation. For several of these variables, the available documentation does not allow a distinction between measurements that were not performed and measurements that were performed clinically but were not accessible in the reviewed record. Consequently, the temporal association supports a dynamic electrocardiographic component but cannot prove causation, and concurrent myocardial injury, electrolyte-driven repolarization abnormality, or artifact cannot be excluded. The patient's hypertrophic cardiomyopathy further limits attribution of all ST-T changes to a single transient mechanism.

## Conclusions

This case offers three transferable points. First, serial bedside electrocardiographic comparison anchored to clinical time points can help distinguish a dynamic pre-QRS dome-and-spike component from fixed baseline abnormalities, such as left ventricular hypertrophy-related repolarization changes. Second, gastric decompression did not abolish the morphology at the short post-decompression interval; the later disappearance occurred only after a confounded multiday period that included improvement in septic shock and gastric distension, vasopressor weaning, and other critical-care interventions. Therefore, neither persistence nor resolution of the sign is diagnostic of causation. Third, although the spiked helmet sign has been described as a marker of critical illness and high mortality risk, this patient stabilized, supporting interpretation of the pattern as a warning sign rather than a deterministic prognosis. When atypical pseudo-ST-segment elevation appears in an unstable patient, reversible thoracoabdominal stressors should be assessed and treated early, while evaluation for acute coronary occlusion, post-resuscitation injury, electrolyte disturbance, and technical artifact proceeds in parallel.
